# Airport noise predicts song timing of European birds

**DOI:** 10.1002/ece3.2357

**Published:** 2016-08-01

**Authors:** Davide M. Dominoni, Stefan Greif, Erwin Nemeth, Henrik Brumm

**Affiliations:** ^1^ Department of Animal EcologyNetherlands Institute of EcologyWageningen6708 PBThe Netherlands; ^2^ Institute of BiodiversityAnimal Health and Comparative MedicineUniversity of GlasgowGlasgowG12 8QQUK; ^3^ Sensory Ecology GroupMax Planck Institute for OrnithologySeewiesen82319Germany; ^4^ Communication and Social Behaviour GroupMax Planck Institute for OrnithologySeewiesen82319Germany; ^5^ BirdLife AustriaVienna1070Austria

**Keywords:** Airport, dawn chorus, ecological novelty, noise, songbirds

## Abstract

Anthropogenic noise is of increasing concern to biologists and medical scientists. Its detrimental effects on human health have been well studied, with the high noise levels from air traffic being of particular concern. However, less is known about the effects of airport noise pollution on signal masking in wild animals. Here, we report a relationship between aircraft noise and two major features of the singing behavior of birds. We found that five of ten songbird species began singing significantly earlier in the morning in the vicinity of a major European airport than their conspecifics at a quieter control site. As birds at both sites started singing before the onset of air traffic in the morning, this suggests that the birds in the vicinity of the airport advanced their activity to gain more time for unimpaired singing before the massive plane noise set in. In addition, we found that during the day, chaffinches avoided singing during airplane takeoffs, but only when the noise exceeded a certain threshold, further suggesting that the massive noise caused by the airport can impair acoustic communication in birds. Overall, our study indicates that birds may be adjusting their mating signals and time budgets in response to aircraft noise.

## Introduction

Environmental noise is known to affect animals in several ways and at different levels of biological organization, ranging from genes and cells to behavior and community assemblage (Kight and Swaddle 2011; McGregor et al. 2013), potentially leading to evolutionary change (Swaddle et al. 2015). In the last decades, researchers have started to assess the impact of a particular form of environmental noise and anthropogenic noise, on individuals and ecosystems (Barber et al. 2010; McGregor et al. 2013). For instance, noise pollution has been linked to changes in sexual signaling in insects (Lampe et al. 2012) and anurans (Sun and Narins 2005), to increased predation in fish (Simpson et al. 2016), and to reduced species richness and altered species interactions in birds (Francis et al. 2009). One of the most studied aspects of anthropogenic noise is its impact on the singing behavior of birds (Gil and Brumm 2014). This impact has strong implications for the evolution of signals as well as for conservation biology. Indeed, as the two main functions of bird song are mate attraction and territory defense (Catchpole and Slater 2008), differences in the efficacy of signal transmission are likely to have major fitness consequences (Swaddle et al. 2015). However, birds possess the behavioral plasticity to mitigate acoustic masking of their songs. In particular, birds increase their vocal amplitude when noise levels rise and, related to this, some birds have also been found to sing at different pitches at locations with high levels of anthropogenic noise (Brumm and Zollinger 2013). In addition, birds are known to adjust the short‐term timing of their songs to avoid overlap with heterospecific songs (Brumm and Zollinger 2013). However, in one of the few experiments conducted on birds, Eurasian wrens (*Troglodytes troglodytes*) did not avoid overlapping their songs with 10‐second bouts of white noise played back to them (Yang and Slabbekoorn 2014). Hence, it is still an open question whether birds use their song timing capacity to adjust their vocal output to short‐term fluctuations of anthropogenic noise.

On a larger timescale, man‐made noise often follows a predictable diurnal pattern, which would theoretically allow birds to shift their song activity away from the noisiest periods. However, only very little is known about whether birds indeed adjust the timing of their singing activity to minimize interference by anthropogenic noise. Male European robins (*Erithacus rubecula*) sing more during the night in urban areas with high daytime noise compared to less noisy locations, suggesting that robins shift their singing behavior from noisy to more quiet hours (Fuller et al. 2007). Moreover, noise pollution during the night can also be linked to an earlier onset of singing activities: High estimates of nighttime noise levels were shown to correlate with an early onset of the dawn song in common blackbirds (*Turdus merula*) (Nordt and Klenke 2013). Another study found that experimental noise exposure before sunrise resulted in earlier singing activity in house sparrows (*Passer domesticus*) and spotless starlings (*Sturnus unicolor*) (but not in four other bird species) (Arroyo‐Solís et al. 2013). In addition, a recent study found that the onset of the dawn song in urban rufous‐collared sparrows (*Zonotrichia leucophrys*) can be predicted by the level of traffic noise later in the day (Dorado‐Correa et al. 2016). The dawn song refers to the marked peak of singing activity around dawn, and because numerous individuals of many bird species join in, this phenomenon is also known as the dawn chorus (Catchpole and Slater 2008). The proximate and ultimate causes of dawn song have been widely studied by behavioral ecologists (Krebs and Kacelnik 1983). While the ultimate aim of dawn song is to attract mates and defend territories (Krebs and Kacelnik 1983), both experimental and theoretical work has suggested that the morning hours are the optimal time for birds to sing (McNamara et al. 1987; Thomas 1999). Recently, Gil and colleagues (Gil et al. 2014) examined the onset of the dawn chorus in areas around several airports in Spain and Germany and found that birds closer to the airports tended to sing earlier. This effect was stronger for those species whose normal onset of the dawn song is relatively late (i.e., closer to the onset of air traffic in the morning), suggesting that the overlap between the dawn chorus and aircraft noise may be crucial for the advancement of singing. However, the study did not relate the timing of the dawn choruses to noise levels, and thus, it is unclear whether noise pollution from air traffic indeed predicts the onset of the dawn chorus.

Another environmental factor related to urbanization, artificial light at night, often covaries with noise levels and might influence some of the aforementioned findings. Indeed, light, and more specifically daylength, is perhaps the strongest environmental cue by which animals, including birds, time their daily and seasonal cycles of activity (Dominoni et al. 2016). Experimental work in captivity (Dominoni et al. 2013a) and semi‐experimental studies in the field have suggested that exposure to artificial light at night can advance the dawn chorus of several songbird species (Kempenaers et al. 2010; Da Silva et al. 2014; Dominoni and Partecke 2015). Thus, it is important to consider such variables in any study that wishes to relate anthropogenic noise to changes in the timing of singing behavior of birds.

The aim of our study was to investigate whether a massive anthropogenic noise source is able to affect the timing of singing of several bird species. We examined two aspects of song timing: First, we recorded the onset of dawn song activity of all bird species singing close to a major European airport (Tegel airport, Berlin) and compared it to control areas of similar habitat, while simultaneously controlling for differences in light levels. Expanding from the work of Gil et al. (2014), we directly tested whether differences in dawn song timing are related to noise levels at dawn or during daytime. Noise around dawn could act as a wake‐up stimulus and thus induce early singing behavior, while prolonged noise during daytime can directly interfere with acoustic communication and force birds to shift their song production to earlier hours, when noise levels are generally low (Fuller et al. 2007). Second, we have conducted targeted song recordings of male chaffinches (*Fringilla coelebs*) during daytime, to test the hypothesis that male birds avoid singing when the massive noise caused by aircraft take‐offs could interfere with song transmission. We predicted that chaffinches sing less often during fly overs by airplanes to avoid acoustic masking of their vocal signals.

## Methods

### Study sites and bird census

The study was conducted in the city of Berlin, Germany, between 23^rd^ and 28th April 2013, and 1st and 4th May 2014. We randomly selected locations in the Jungfernheide forest adjacent to Tegel airport and control forest locations in the Tegeler Forst (see Supporting Information). Tegel airport operates between 0600 and 2300 h with airplane take‐offs about every 2 min (http://www.berlin-airport.de/en/travellers-txl/arrivals-and-departures/departures/index.php). Few postal and special authorization flights may be conducted during the flight ban between 2300 and 0600 h, but during our study, we heard no plane taking off in the morning before 0600 h.

**Figure 1:**
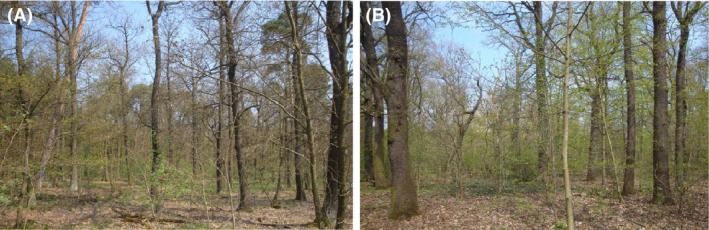
Examplary point locations at the airport site (A) and the control site (B). Both sites were forests with an area of several square kilometers located within the city of Berlin and both are protected landscapes under German nature protection law (area numbers Berlin LSG‐02, Berlin LSG‐28).

We compared the onset of the dawn chorus at 14 locations in the Jungfernheide forest adjacent to Tegel airport (N52°33′43′′, E13°15′43′′) with 14 control locations in the Tegeler Forst (N52°35′34′′, E13°14′45′′). Both forests are of similar age and structure, with oak (*Quercus petraea* and *Q. robur*) and pine trees (*Pinus sylvestris*) as the dominant tree species (Fig. 1). The census locations were chosen randomly within each site but spaced at least 150 m apart and not closer than 100 m to the forest edge to avoid light pollution from surrounding urban areas. The airport locations were within 430–1190 m from the runway, whereas the control locations were more than 4 km away from it.

### Dawn song recordings

At least 1 h before the beginning of civil twilight, four researchers experienced with bird songs took position at four different locations, distributed among the airport and forest site. No bird was heard singing at the time we arrived at the sites, so we assumed that all birds started singing within the hour before the onset of twilight. We noted the time when the first song of a species was heard. Due to logistic constraints, additional locations were equipped with audio recorders instead of human observers: two control locations on the last day of the study period in 2013 (April 28th) and at one airport and two control locations in 2014. In 2013, we used two digital audio recorders (TASCAM TR‐08, Marantz PMD 660) connected to an omnidirectional microphone (T‐BONE EM‐9600 or Sennheiser ME 62) positioned at a height of two meters. In 2014, only one set of equipment was used (Marantz PMD 660 with a Sennheiser ME 62 microphone). All audio recordings were made with a sample rate of 44.1 kHz, and they were done from at least 1 h before the beginning of civil twilight until at least 0630 h. Audio recordings were subsequently screened for the onset of singing activity for each species in the same way it was performed in the field by the human observers. In 2014, only one researcher took position in the forest. When this human observer was deployed at an airport site, an audio recorder was placed at a control site and vice versa. In total, the onset of singing activity was monitored at 14 airport and 14 control locations, each of which was sampled once. Thirty‐three species were recorded, 31 of which were present at both sites. Of these 31 species, we analyzed those ten for which we had records from at least ten different locations per site (Table S1).

### Noise and light recordings

We measured nighttime and daytime noise levels with two digital sound level meters (PCE‐353, Casella CEL‐24X). Noise measurements were taken at several locations of both sites on the same day as the dawn song at each respective location. First, we measured the noise at 0500 h, approximately the time of onset of civil twilight during the period of our study, by deploying the digital sound level meters at a height of 1.6 m with the measuring microphone pointing upward, as maximum sound level (L_AF,_ dB re. 20*μ*Pa) of a one‐minute measuring interval. In 2014, noise measurements at dawn were taken only for one location each day. Daytime noise was measured at all recording locations between 06:00 and 09:00 h as the maximum L_AF_ of a five‐minute interval (i.e. capturing at least one airplane take‐off).

To characterize the nocturnal light environment at both sites and check for the presence of light pollution, which could have biased the interpretation of the results, light intensity was measured using a LI‐210 photometer sensor attached to a LI‐1400 data logger (LI‐COR, Lincoln, NE, USA). The LI‐210 sensor measures light with the same sensitivity as the typical human eye and it is commonly used to measure both interior and exterior artificial lighting. Light measurements were taken for 1 min approx. 1.6 m above and parallel to ground with the light sensor pointing upward, similar to the procedure used in Dominoni et al. (2014). Light recordings were performed at the same time and places as noise measurements, but only in 2013.

### Daytime song recordings

To assess whether birds adjust the timing of their songs to avoid overlap with fluctuating anthropogenic noise, we recorded singing chaffinches (*Fringilla coelebs*) at the airport site during plane take‐offs. We chose chaffinches for several reasons: They occurred in high numbers at the airport site; they sang regularly during the day; they could be approached to a few meters without ceasing to sing; and finally their song has been well studied, including song performance in noise (Riebel et al. 2015). In particular, it has been found that chaffinches sing with increased serial redundancy in noisy habitats: Males close to noisy streams repeat a song type more often before switching to a new one than those in quieter areas, a behavior that will help to maintaining signal transmission in noise (Brumm and Slater 2006).

Seven males were recorded between 25th and 28th April 2013 and ten males between 1st and 4th May 2014, between 0825 and 1240 h. Each bird was recorded at a different location with at least 150 m between them. The audio recordings were made from a distance between 5 to 15 m to the singing bird, using a solid‐state recorder (Marantz PMD 660, sample rate 44.1 kHz) and an omnidirectional microphone (Sennheiser ME 62). At the same time, the noise level of each plane take‐off was recorded as maximum L_AF_ (dB re. 20 *μ*Pa) using a Casella CEL‐24X SPL meter. The onset time and the duration of the songs and plane noises were measured to the nearest 10 msec in oscillograms of the audio recordings produced in Avisoft SASLab Pro (version 5.2.08) (Avisoft Bioacoustics, Glienicke, Germany). The duration of the plane noise was determined as the period during which the noise amplitude dropped to 18 dB below the peak amplitude. This threshold was chosen because (1) it was well above the natural background noise in the plane recording with the lowest peak amplitude and (2) it yielded similar durations for all aircraft noises in our sample (mean ± SD: 29 ± 3.1s). For the analysis, we used only bouts of continuous singing, which are considered to be with silent intervals between songs shorter than 30 sec in chaffinches (Slater 1983), but the periods of aircraft noise were excluded from this criterion. Using this standard reduced the sample size to 15 males. On average, the analyzed recordings had a duration of 8.8 min per male (range: 4.6–17.8 min) during which time the birds sang 54.7 songs (range 27–111) and 4 planes (range. 3–8) took off.

### Statistical analyses

Statistical analyses were conducted with software R 2.15.1 (R Development Core Team, 2011) and Matlab 12.1 (“MATLAB 8.0 and Statistics Toolbox 8.1 [Link]). All tests were two‐tailed, and we applied a significance level *α *= 0.05.

The overall effect of site (airport vs. control) and daytime noise on the onset of dawn song of all recorded species was analyzed with two linear mixed models (LMMs) with the locations included as random factors and species and year as fixed factor. The significance of the two models was tested with likelihood‐ratio tests, comparing each model to null models that considered year and species but did not include site or daytime noise as predictors. As a second step, we ran two independent LMMs for each species to test for differences in song onset between sites and for variation of song onset with daytime noise levels (Table S2).

We used a randomization procedure to examine whether chaffinches avoided overlapping the noise from taking off airplanes. The procedure was custom‐coded using Matlab (The MathWorks, Inc. Ismaning, Germany) (for details see, Brumm (2006)). In brief, the test compared the observed temporal overlap between songs and noise with a chance distribution generated by 10,000 randomizations. First, we ran one global test summing over all individuals and, subsequently, we conducted individual test for each bird. For each recording, the chaffinch songs were randomized with respect to time of onset and the overlap with the aircraft noise was then compared to the observed overlap. As chaffinches are discontinuous songsters (i.e., they do not produce two songs without a pause between them), the randomization was constrained such that there was always a pause of at least 1 sec between songs (0.96 sec is the shortest intersong interval found in a set of recordings from over 100 birds (Brumm et al. 2009a,b)).

## Results

At the onset of dawn, noise levels did not differ significantly between airport and control sites (*z* = −1.19, *P* = 0.236). Likewise, light levels were not significantly different between the two sites (*z* = 0.01, *P* = 0.991). In contrast, daytime noise levels were on average 30 dB(A) higher at the airport than the control locations due to the noise pollution from airplane take‐offs and landings (*z* = −9.89, *P* < 0.001). Table 1 displays the minimum, maximum, and median values for light intensity and noise measurements.

**Table 1:** Differences in dawn (0500 h) and daytime (0600–0900 h) levels of noise and light intensity between the airport and control locations. Dawn measures: N_airport_ = 8, N_control_ = 5; daytime noise level: N_airport_ = N_control_ = 14

		Dawn light intensity (lux)	Dawn noise level [dB(A) SPL]	Daytime noise level [dB(A) SPL]
Airport	min	0.00014	40	70
	median	0.00018	46	78
	max	0.004	60	87
Control	min	0.0002	41	42
	median	0.0003	45	48
	max	0.0004	51	59

**Figure 2:**
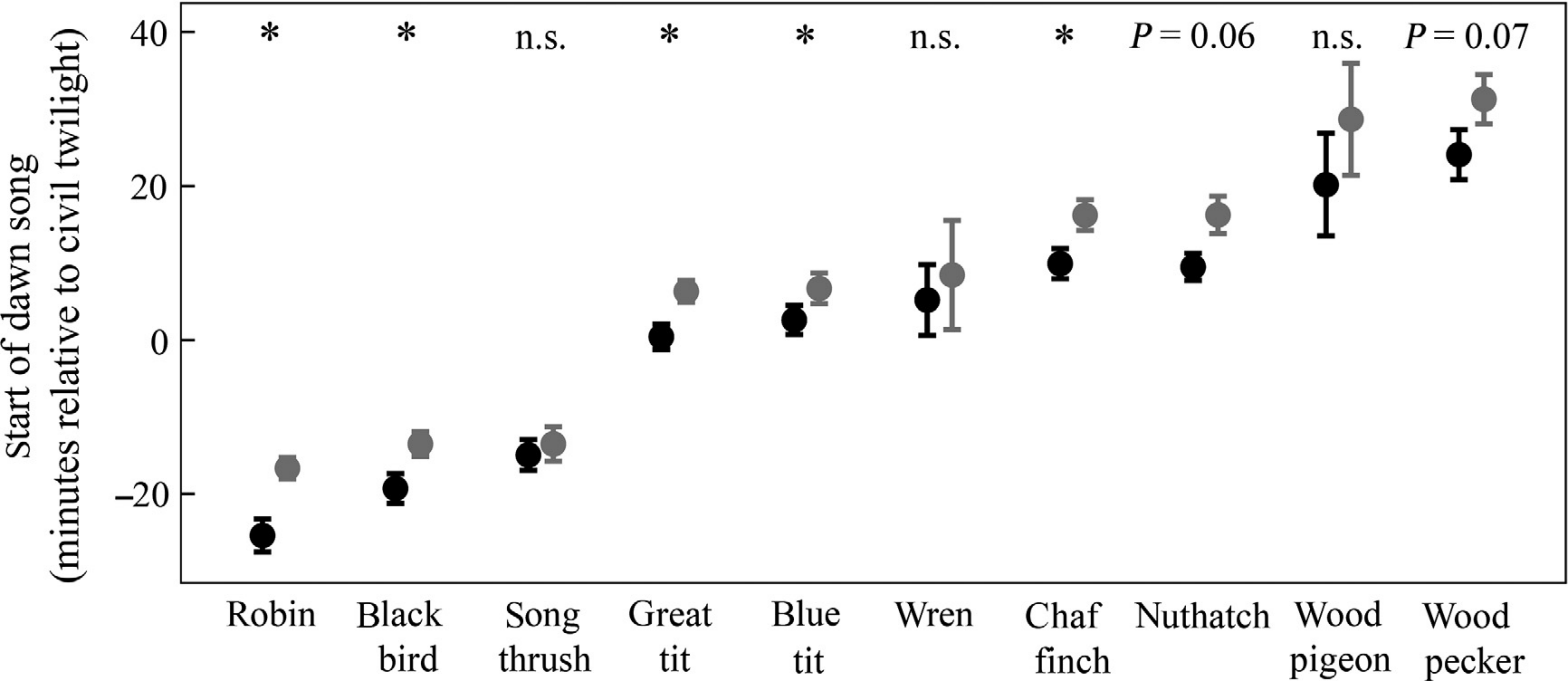
Onset of the dawn song at Tegel airport (black) and control locations (gray). Dawn song times are given as means ± SE. (*: *P *< 0.05, n.s.: not significant, see Table 2)

The time at which birds started to display dawn chorus varied between species. European robins were the earliest singers and began to sing on average 20 min before dawn, while great spotted woodpeckers were the latest, 25 min after dawn (Fig. 2). The dawn chorus onset varied with daytime noise levels (likelihood‐ratio test: log‐likelihood null model = 53.5, log‐likelihood model = 56.0, df = 1, chi^2^ = 3.46, *P* = 0.03, Fig. 2) and, correspondingly, birds started to sing earlier at the airport site compared to the control site (likelihood‐ratio test: log‐likelihood null model = 53.5, log‐likelihood model = 56.5, df = 1, chi^2^ = 5.91, *P* = 0.02). However, both analyses indicated that the difference in the onset time of the dawn song also depended on the species considered (see Supporting Information). Independent LMMs ran for each species showed that European robins, common blackbirds, blue tits, great tits, and chaffinches sang significantly earlier at the airport site compared to the control site; the Eurasian nuthatch and the great spotted woodpecker showed a trend in the same direction (Table 2). In all of these species, higher levels of daytime noise were significantly associated with earlier dawn song onsets (or showed a statistical trend in this direction), except for the woodpecker.

**Table 2:** Outcomes of linear mixed models testing the effects of the recording site (airport vs. control) and the daytime noise level on the onset of the dawn chorus of a species (estimate ± standard error, *t*‐value and *P*‐value, see Table S2 for details)

Species	Site	Daytime noise
European robin	*e* = −8.8 ± 2.50, *t* = −3.52,	*e* = −0.26 ± 0.08, *t* = −3.09, *P *= 0.005
European blackbird	*e* = −5.8 ± 2.44, *t* = −2.37, *P *= 0.026	*e* = −0.19 ± 0.08, *t* = −2.45, *P *= 0.022
Song thrush	*e* = −1.43 ± 3.02, *t* = −0.47, *P *= 0.641	*e* = −0.07 ± 0.09, *t* = −0.68, *P *= 0.506
Great tit	*e* = −5.74 ± 1.70, *t* = −3.37, *P *= 0.003	*e* = −0.15 ± 0.6, *t* = −2.59, *P *= 0.016
blue tit	*e* = −4.08 ± 1.77, *t* = −2.29, *P *= 0.031	*e* = −0.11 ± 0.06, *t* = −1.93, *P *= 0.066
Eurasian wren	*e* = −0.97 ± 7.89, *t* = 0.12, *P *= 0.903	*e* = −0.18 ± 0.24, *t* = 0.73, *P *= 0476
Common chaffinch	*e* = −6.29 ± 2.4, *t* = −2.62, *P *= 0.015	*e* = −0.16 ± 0.08, *t* = −1.93, *P *= 0.066
Eurasian nuthatch	*e* = −6.59 ± 3.26, *t* = −2.02, *P *= 0.06	*e* = −0.15 ± 0.11, *t* = −1.37, *P *= 0.194
Wood pigeon	*e* = −4.17 ± 9.12, *t* = −0.48, *P *= 0.652	*e* = −0.15 ± 0.31, *t* = −0.48, *P *= 0.634
Great spotted woodpecker	*e* = −7.45 ± 3.94, *t *= −1.89, *P *= 0.07	*e* = −0.19 ± 0.13, *t* = −1.40, *P *= 0.175

**Figure 3:**
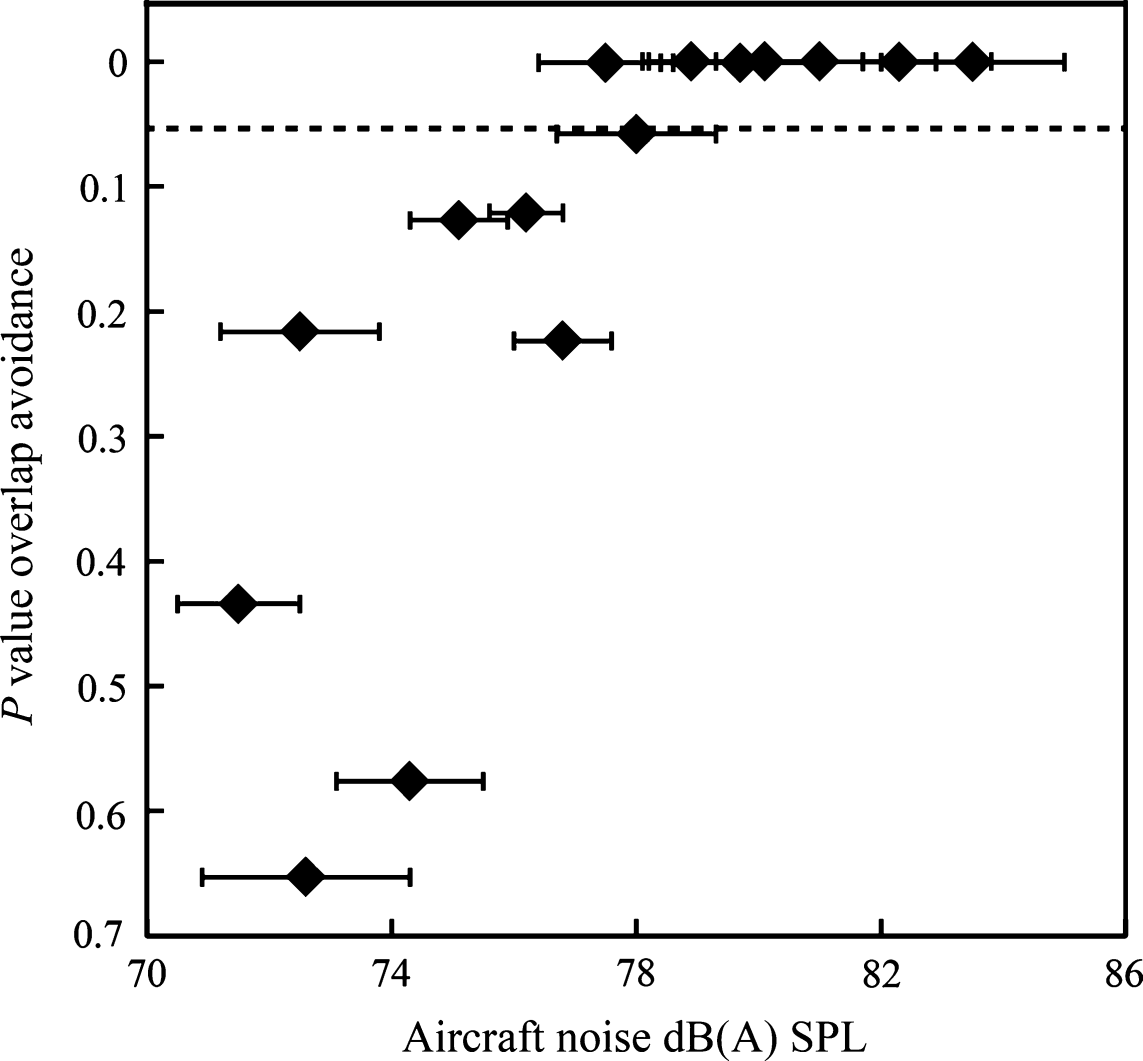
Adjustment of chaffinch song patterning in relation to the mean sound level (± SD) of airplane noise (re. 20 *μ*Pa). *P* values are based on randomization tests (see Methods for details), values smaller than 0.05 (dotted line) indicate a lesser percentage of song during airplane noise than expected by chance.

Moreover, we found that the level of aircraft noise did not only predict the onset of the dawn chorus but also affected the short‐term timing of songs during daytime singing. Chaffinches near the airport sang much less often during aircraft take‐offs than predicted by chance (10,000 simulations, *P* < 0.0001). However, the avoidance of song overlap was strongly related to the sound level of the aircraft noise: birds avoided to sing during noise bouts with a maximum amplitude above approx. 78 dB(A). Below this threshold, they did not change their song pattern significantly (Fig. 3).

## Discussion

Aircraft and airport sites have long been suggested to affect wildlife, especially birds, due to collision‐induced mortality, the stress that high noise levels can produce, and the interference with acoustic communication (Burger 1985; McGregor et al. 2013). In this study, we document a relationship between the temporal variation of singing behavior and the presence of airplane noise. We discovered that songbird species in the vicinity of Tegel airport in Berlin advanced the onset of their dawn song compared to conspecifics singing in a nearby forest which was less affected by aircraft noise. The differences in dawn song onset in our study were between 5 and 10 min, thus smaller advances than those reported in other studies on dawn chorus shifts in relation to noise and/or light pollution (Kempenaers et al. 2010; Nordt and Klenke 2013; Da Silva et al. 2014; Dominoni et al. 2014; Gil et al. 2014). However, even a small difference in the onset of dawn song can be crucial for the reproduction of birds: Male blue tits (*Cyanistes caeruleus*) that advanced their song activity at dawn by only 5 min were found to have more mating partners and were more likely to gain extra‐pair paternity (Poesel et al. 2006; Kempenaers et al. 2010). A recent study has shown that birds in the vicinity of airports start singing earlier in the morning (Gil et al. 2014), and taken together with our noise measurements, the overall picture suggests a general effect of airport noise on song timing in birds. Because we measured daytime noise at several locations across our sites, our data allows, for the first time, to directly correlate variation in aircraft noise to the advance in the onset of dawn chorus. In addition, we have shown that noise levels did not differ between the airport and the control forest at dawn, before the onset of airplane traffic. Thus, our findings suggest that the difference in dawn chorus timing were related to aircraft noise later during the day. This notion of birds anticipating interference by noise is corroborated by another study that found that traffic noise later in the day predicts the onset of the dawn chorus in a tropical songbird (Dorado‐Correa et al. 2016). However, experiments are necessary to establish a causal link between anthropogenic noise and changes in bird behavior (Nemeth and Brumm 2009). To this aim, approaches like that used by Barber and collaborators (Ware et al. 2015), who deployed an array of speakers in the middle of a forest to act as a “phantom road,” may be the way forward.

As in previous studies on the impact of anthropogenic noise or light on dawn chorus singing (Kempenaers et al. 2010; Da Silva et al. 2014), we found an effect in several but not all species of a community. It has been suggested that anthropogenic noise should affect the dawn song onset more strongly in species which start singing later in the morning, that is closer to the start of human activities (Da Silva et al. 2014; Gil et al. 2014). However, our data only partially support this notion: We found that not only late species advanced their dawn song onset in relation to daytime noise levels, but also early species, which start singing before the onset of civil twilight. Indeed, the biggest shift was observed in the robin, the species that began singing the earliest. Interestingly, this is also the species that has previously been shown to sing more often during the night in areas with high levels of daytime noise (Fuller et al. 2007). While the time of initiation of the dawn chorus in passerine birds has received considerable attention, we know much less about when singing behavior ends in the morning (Catchpole and Slater 2008). We suggest that early singing birds do not necessarily end their dawn chorus before later risers, and therefore, they might be equally affected by high daytime noise levels.

Several studies have shown that increased light intensity at night can make birds advance their dawn song (Kempenaers et al. 2010; Dominoni et al. 2013a, 2014). To minimize such light effects in this study, we took our measurements at several locations within the two forests, thereby reducing a potential influence of within‐forest differences in vegetation structure on light levels. Hence, light intensity at dawn did not differ significantly between the airport and control locations. If anything, the light level at dawn was slightly, although not significantly, higher in the control areas than at the airport locations, which means that any effects of light advancing dawn song would have reduced the observed effect of noise on dawn chorus onset. As the two forests were only a few kilometers apart, it is unlikely that other factors that may modulate light intensity, such as sky glow (Kyba et al. 2011), could have had an impact. As we have not measured light pollution during the months before the start of the study, we cannot exclude that small differences in the nocturnal light environment before we conducted our measurements could have affected our findings. However, we think this is a highly unlikely scenario because (1) the forest structure of our control and airport locations were very similar to each other (Fig. 1), (2) all sampling points were located more than 100 m from the forest edge and thus were not considerably affected by light pollution, and (3) there is no reason to assume that light pollution regimes have changed before we conducted the study. Overall, our results do not contradict previously reported effects of artificial light at night on dawn song timing, but rather they suggest that also anthropogenic daytime noise can advance dawn singing. Similarly, other unmeasured environmental factors that might differ between the two forests could have affected the observed results. However, we believe this to be highly unlikely as the two forest sites were very close to each other and very similar in their structure, thus factors such as temperature, cloud cover, and parasite communities are not likely to differ between them. We cannot exclude this possibility though, and we call for further experimental work. Moreover, although we sampled 14 locations at each site, our results are based on noise pollution from only one particular airport. However, the pattern of birds starting to sing earlier close to heavily noise polluted sites seems to be widespread, as indicated by data from other airports (Gil et al. 2014).

Several behavioral and physiological mechanisms may account for the observed differences in dawn song timing. Males may benefit from singing early, as advancing their dawn song can improve their reproductive success by securing more extra‐pair offspring (Poesel et al. 2006; Kempenaers et al. 2010). We speculate that early singing males reallocate the time for song production to quieter times of the day, when they are more likely to be heard by conspecifics (Brumm and Zollinger 2013). Such behavioral plasticity could be adaptive when it results in a higher success of territorial defense, mate guarding, and/or extra‐pair paternity. If this is true, environmental selection for faster circadian clocks could be accounted for the earlier onset of dawn chorus (Dominoni et al. 2013b). Alternative explanations would be learning or behavioral plasticity. Plasticity seems less likely, though, as the birds changed their behavior in anticipation of the environmental change and no external cues are known that would allow them to predict this change (Gil et al. 2014). In addition to elucidating the mechanism of the observed shifts in the dawn chorus, we also suggest that future studies focus on reproductive benefits, as well as on the potential costs, of this phenomenon. This is particularly interesting because birds in noisy areas may suffer from decreased reproductive success (Kight et al. 2012; Halfwerk and Slabbekoorn 2014). Therefore, it would be worthwhile to examine different aspects of reproductive behavior while controlling for extra‐pair paternity rate, to fully assess the reproductive consequences of breeding in noisy environments.

Our findings suggest the possibility that not only the onset time of the dawn chorus is important for birds but also the total duration of unmasked dawn singing. As the airport locations were not noise polluted at dawn, the advancement of the dawn chorus may allow the birds to sing for a longer time before the aircraft noise set in. Once air traffic has commenced in the morning, acoustic communication in areas around airports is heavily constrained. In the case of Tegel airport, noise pollution occurred with take‐offs about every 2 min, producing noise levels of up to 87 dB(A) in bird habitats. We found that chaffinches ceased singing when peak noise levels of planes exceeded approx. 78 dB(A). Given that the aircraft noise bouts had durations of about 30 sec, this means that during air traffic operations about 25% of potential signaling time is lost in bird territories near the airport. In places with lower noise levels, where birds kept on singing during fly overs, the active space of these songs will be reduced due to acoustic masking (Dooling and Blumenrath 2013). As song rate is important for birds for both male–male competition and mate attraction (Catchpole and Slater 2008), a reduction of singing time or reduced audibility of songs can affect territorial behavior and reproductive success. However, the potential fitness consequences of airplanes drowning out chaffinches and other birds are still unexplored. At aircraft peak amplitudes below approx. 78 dB(A), the chaffinches in our study did not significantly reduce their song overlap with the noise. This is in line with a previous study by Yang and Slabbekoorn (2014) that used white noise with an average amplitude of 64 dB(A). Considering that white noise at this sound level can stimulate birds to increase their vocal output (Brumm et al. 2009a,b; Brumm and Zollinger 2013), it is not surprising that the previous study did not find a reduction of singing activity during the noise exposure. At higher amplitudes, however, white noise can induce a reduction or even a complete cessation of song in captive birds (Brumm and Zollinger 2013), which is consistent with our findings from chaffinches and airplane noise. On a proximate level, intense noise may act as an aversive stimulus, suppressing song production. In functional terms, the birds escaped masking of their sexual signals by avoiding temporal overlap with high‐amplitude airplane noise.

In conclusion, our study offers a new perspective on the effects of anthropogenic noise on the behavior of birds, indicating that birds may be adjusting their mating signals and time budgets in response to intense anthropogenic noise, both on the level of circadian rhythms and the level of short‐term responses to fluctuating noise levels. Such individual adjustments to ecological novelty have the potential to affect the fitness of the singer and thus, in the long‐term, might even change population dynamics.

## Conflict of Interests

We declared that we have no conflict of interests.

## Supporting information


**Table S1.** Bird species detected in acoustic censuses conducted in a forest at Tegel airport and a control forest 4 km away from the airport.
**Table S2.** Species‐specific results of linear mixed models with the dawn song onset in relation to the beginning of civil twilight as predicted variable and daytime noise levels or site as predictor variables (*n* = 28).
