# Supplementary material for: Airport noise predicts song timing of European birds
Source: Ecol Evol. 2016 Aug 1;6(17):6151–9. doi: 10.1002/ece3.2357 (PMC5016638; doi:10.1002/ece3.2357)
Supplement: Supplementary file 1 — Table S1. Bird species detected in acoustic censuses conducted in a forest at Tegel airport and a control forest 4 km away from the airport. Table S2. Species‐specific results of linear mixed models with the dawn song onset in relation to the beginning of civil twilight as predicted variable and daytime noise levels or site as predictor variables (n = 28). [file ECE3-6-6151-s001.docx]

**Electronic Supplementary Material**

**Airport noise predicts song timing of European birds**

Davide M. Dominoni, Stefan Greif, Erwin Nemeth and Henrik Brumm

***Bird communities at the study sites***

**Table S1.** Bird species detected in acoustic censuses conducted in a forest at Tegel airport and a control forest 4 km away from the airport. The censuses were based on point counts lasting from 45 minutes before dawn until 0600 hours. The time of day was recorded for the first song (or the drumming of woodpeckers, respectively) heard at each location. Species that were detected at a minimum of ten point locations at each site were included in the dawn chorus analysis (indicated in bold). In total, 14 point counts were made at both sites.

| Species | Number of AIRPORT locations with occurrence | Number of CONTROL locations  with occurrence |
| --- | --- | --- |
| Black woodpecker (*Dryocopus martius*) | 5 | 3 |
| Blue tit (*Cyanistes caeruleus*) | **13** | **13** |
| Coal tit (*Periparus ater*) | 3 | 3 |
| Common blackbird (*Turdus merula*) | **14** | **14** |
| Common chaffinch (*Fringilla coelebs*) | **14** | **14** |
| Common chiffchaff (*Phylloscopus collybita*) | 2 | 2 |
| Common cuckoo (*Cuculus canorus*) | 5 | 2 |
| Common nightingale (*Luscinia megarhynchos*) | 4 | 2 |
| Common redstart (*Phoenicurus phoenicurus*) | 2 | 2 |
| Common starling (*Sturnus vulgaris*) | 1 | 1 |
| Eurasian blackcap (*Sylvia atricapilla*) | 6 | 8 |
| Eurasian jay (*Garrulus glandarius*) | 5 | 3 |
| Eurasian nuthatch (*Sitta europea*) | **10** | **12** |
| Eurasian treecreeper (*Certhia familiaris*) | 9 | 8 |
| Eurasian wren (*Troglodytes troglodytes*) | **10** | **11** |
| European crested tit (*Lophophanes cristatus*) | 1 | 3 |
| European pied flycatcher (*Ficedula hypoleuca*) | 4 | 2 |
| European robin (*Erithacus rubecula*) | **13** | **14** |
| Great spotted woodpecker (*Dendrocopos major*) | **13** | **14** |
| Great tit (*Parus major*) | **14** | **13** |
| Green woodpecker (*Picus viridis*) | 2 | 3 |
| Hawfinch (*Coccothraustes coccothraustes*) | 5 | 3 |
| Hooded crow (*Corvus cornix*) | 6 | 6 |
| Lesser whitethroat (*Sylvia curruca*) | 0 | 1 |
| Mallard (*Anas platyrhynchos*) | 2 | 5 |
| Marsh tit (*Poecile palustris*) | 1 | 0 |
| Middle spotted woodpecker (*Dendrocopos medius*) | 2 | 2 |
| Northern raven (*Corvus corax*) | 1 | 0 |
| Short-toed treecreeper (*Certhia brachydactyla*) | 2 | 3 |
| Song thrush (*Turdus philomelos*) | **14** | **14** |
| Tawny owl (*Strix aluco*) | 2 | 2 |
| Willow warbler (*Phylloscopus trochilus*) | 3 | 3 |
| Wood pigeon (*Columba palumbus*) | **11** | **13** |
| Wood warbler (*Phylloscopus sibilatrix*) | 6 | 9 |

***Variation in dawn song onset***

**Table S2.** Species-specific results of linear mixed models with the dawn song onset in relation to the beginning of civil twilight as predicted variable and daytime noise levels or site as predictor variables (n=28). Site: airport or control site, daytime noise: maximum SPL measured in a five-minute interval between 0600 and 0900 hours.

| Species | Variable | Estimate | Std. error | t-value | p-value |
| --- | --- | --- | --- | --- | --- |
| European robin | intercept  daytime noise  year | -5.45  -2.57  4.02 | 5.45  0.08  3.13 | -1.00  -3.09  1.28 | 0.33  0.005  0.21 |
|  | intercept  site  year | -17.47  -8.81  3.87 | 1.85  2.50  3.01 | -9.44  -3.52  1.29 | < 0.0001  0.002  0.21 |
| Common blackbird | intercept  daytime noise  year | -5.20  -0.19  4.95 | 5.16  0.08  2.95 | -1.01  -2.45  1.67 | 0.32  0.02  0.11 |
|  | intercept  site  year | -14.52  -5.79  4.74 | 1.84  2.44  2.97 | -7.89  -2.37  1.60 | < 0.0001  0.03  0.12 |
| Song thrush | intercept  daytime noise  year | -9.87  -0.07  -0.72 | 6.41  0.10  3.67 | -1.54  -0.68  -0.20 | 0.14  0.51  0.85 |
|  | intercept  site  year | -13.33  -1.43  -0.79 | 2.28  3.02  3.68 | -5.85  -0.47  -0.22 | < 0.0001  0.64  0.83 |
| Blue tit | intercept  daytime noise  year | 9.99  -0.12  14.36 | 4.03  0.06  2.53 | 2.48  -1.93  5.67 | 0.02  0.07  < 0.0001 |
|  | intercept  site  year | 4.49  -4.08  14.30 | 1.31  1.78  2.46 | 3.42  -2.29  5.81 | 0.002  0.031  < 0.0001 |
| Great Tit | intercept  daytime noise  year | 11.01  -0.15  8.96 | 3.89  0.06  2.19 | 2.83  -2.59  4.08 | 0.007  0.02  0.0004 |
|  | intercept  site  year | 4.29  -5.74  8.74 | 1.31  1.70  2.04 | 3.27  -3.37  4.28 | 0.003  0.003  0.0003 |
| Eurasian wren | intercept  daytime noise  year | -9.14  0.18  25.69 | 16.25  0.25  9.87 | -0.56  0.73  2.60 | 0.58  0.48  0.02 |
|  | intercept  site  year | 1.78  0.97  24.48 | 5.98  7.89  10.04 | 0.30  0.12  2.44 | 0.77  0.90  0.03 |
| Common chaffinch | intercept  daytime noise  year | 21.00  -0.16  9.83 | 5.39  0.08  3.08 | 3.90  -1.93  3.19 | 0.0006  0.07  0.004 |
|  | intercept  site  year | 14.14  -6.29  9.67 | 1.81  2.40  2.93 | 7.81  -2.62  3.30 | < 0.0001  0.02  0.003 |
| Eurasian nuthatch | intercept  daytime noise  year | 22.43  -0.15  3.77 | 7.29  0.11  8.10 | 3.08  -1.34  0.47 | 0.006  0.20  0.65 |
|  | intercept  site  year | 16.09  -6.59  1.91 | 2.25  3.26  7.79 | 7.15  -2.02  0.25 | < 0.0001  0.06  0.81 |
| Wood pidgeon | intercept  daytime noise  year | 28.5862  -0.1453  31.0521 | 19.6621  0.3028  12.1566 | 1.454  -0.480  2.554 | 0.1608  0.6364  0.0185 |
|  | intercept  site  year | 21.537  -4.174  31.007 | 6.689  9.124  12.198 | 3.220  -0.457  2.542 | 0.004  0.65  0.019 |
| Great spotted woodpecker | intercept  daytime noise  year | 36.25  -0.19  14.75 | 8.63  0.13  4.88 | 4.20  -1.40  3.02 | 0.0003  0.17  0.006 |
|  | intercept  site  year | 28.17  -7.45  14.56 | 2.91  3.96  4.73 | 9.67  -1.89  3.08 | < 0.0001  0.07  0.005 |
